# Recent Advances on Cyan‐Emitting (480 ≤ *λ *≤ 520 nm) Metal Halide Perovskite Materials

**DOI:** 10.1002/smsc.202000077

**Published:** 2021-03-08

**Authors:** Yueyue Shen, Chuanzhong Yan, Kebin Lin, Yaping Zhao, Shanrong Xu, Biao Zhou, Zhanhua Wei, Keyou Yan

**Affiliations:** ^1^ School of Environment and Energy State Key Laboratory of Luminescent Materials and Devices National Engineering Laboratory for VOCs Pollution Control Technology and Equipment South China University of Technology Guangzhou 510006 China; ^2^ Institute of Luminescent Materials and Information Displays College of Materials Science and Engineering Huaqiao University Xiamen 361021 China

**Keywords:** cyan-emitting perovskites, light-emitting diodes, metal halide, perovskites

## Abstract

Over the past several years, perovskite‐based luminescent materials and devices have attracted considerable research interest and achieved superior performance, including red/near‐infrared, green, and blue regions. Despite the abundant research progress in the above‐mentioned luminous regions, studies on cyan‐emitting perovskites are still lacking. However, cyan‐emitting perovskite materials are of great importance and have many promising applications, especially for high‐quality lighting and light communication. Herein, the recent research progress on perovskite with cyan emission is summarized, including the preparation methods and improvement on device performance. The preparation strategies are categorized into compositional engineering, dimensional engineering, and size engineering. The corresponding device performance is displayed too. Furthermore, the strategies of performance enhancement and future perspectives are proposed in the end. There is hope that this minireview can trigger more attention to this particular emitting region.

## Graphical abstract

Cyan‐emitting perovskite materials show great potentials in various applications. Preparation approaches for stable cyan emission are summarized as compositional engineering, dimensional engineering, and size engineering. The corresponding light‐emitting diodes (LEDs) device performance and strategies for improvement are presented.

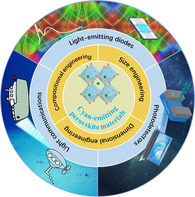

## 1 Introduction

Recently, metal halide perovskites are emerging as fantastic optoelectronic materials. The metal halide perovskite adopts the ABX_3_ structure, where A represents monovalent cation (e.g., CH_3_NH_3_^+^, CH(NH_2_)_2_^+^, Cs^+^…), B is a divalent cation (e.g., Pb^2+^, Sn^2+^…), and X is a monovalent halide or pseudohalide anion (e.g., Cl, Br, I, SCN…).^[^ ^1^, ^2^ ^]^ The first organic–inorganic hybrid perovskite was first discovered by Weber in 1978.^[^ ^3^ ^]^ In 2009, Miyasaka's group used the hybrid perovskite as a visible‐light sensitizer to fabricate liquid dye‐sensitized solar cells. This work is widely known as the first report of perovskite solar cells (PSCs).^[^ ^4^ ^]^ In the following years, the liquid electrolyte was replaced with solid transporter, and the PSCs have proved to be a game changer in the photovoltaic field. The development of solid‐state PSCs also triggered the research effort on light‐emitting diodes (LEDs),^[^ ^5^ ^]^ semiconductor lasers,^[^ ^6^, ^7^, ^8^ ^]^ display components,^[^ ^9^ ^]^ and light communication.^[^ ^10^ ^]^ The perovskite‐based luminescent materials are becoming more and more attractive owing to their excellent properties, such as tunable emission wavelength,^[^ ^11^ ^]^ high‐color purity,^[^ ^12^ ^]^ low cost,^[^ ^13^ ^]^ and high photoluminescence quantum yield (PLQY).^[^ ^14^ ^]^ The first report of room‐temperature operative perovskite LEDs (PeLEDs) was released by Friend and co‐workers in 2014, and the device performance was not so good at the time.^[^ ^15^ ^]^ To date, high external quantum efficiency (EQE) of green‐(≥20%),^[^ ^16^, ^17^ ^]^ red‐(≥20%),^[^ ^18^ ^]^ near‐infrared‐(≥20%),^[^ ^19^, ^20^ ^]^ and (deep) blue‐(>10%)^[^ ^21^ ^]^ emitting perovskites has been achieved.

**Figure 1:**
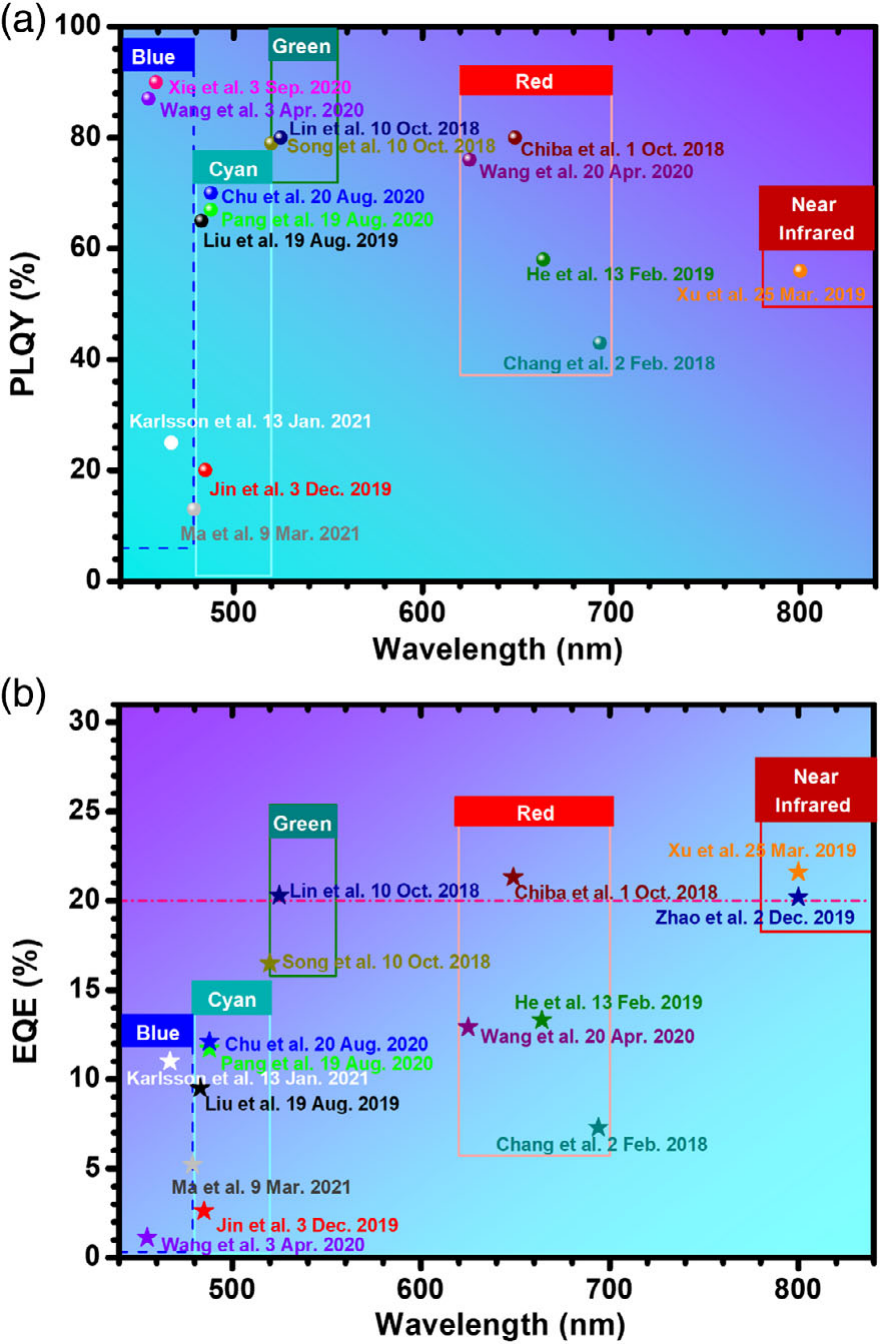
The development of cyan‐emitting perovskites is behind their counterparts. a) PLQY comparison of the typical perovskite light‐emitting materials. b) EQE comparison of the typical PeLEDs performance (the same colors of spheres/pentagrams and captions represent the results collected from the same reference; also, data shown in this figure are collected from the representative research works, respectively^[^ ^16^, ^18^, ^19^, ^20^, ^21^, ^22^, ^23^, ^41^, ^63^, ^72^, ^73^, ^74^, ^75^, ^76^, ^77^, ^78^, ^79^, ^80^ ^]^).

However, the development of cyan‐emitting (480 ≤ *λ *≤ 520 nm) PeLEDs is far behind the regions mentioned earlier and receives much less attention. First, as shown in **Figure** **1**a, the reported PLQY values of cyan‐emitting perovskites are much less than that of the green‐(520–555 nm), red‐(620–700 nm), and deep blue‐(440–479 nm) ones. Second, the EQE of the PeLEDs is still far less than the expected values (Figure 1b). For example, the highest EQE of cyan PeLED (12.1%)^[^ ^22^ ^]^ is significantly lower than the average performance (≈20%) of other color‐emitting perovskites.

**Figure 2:**
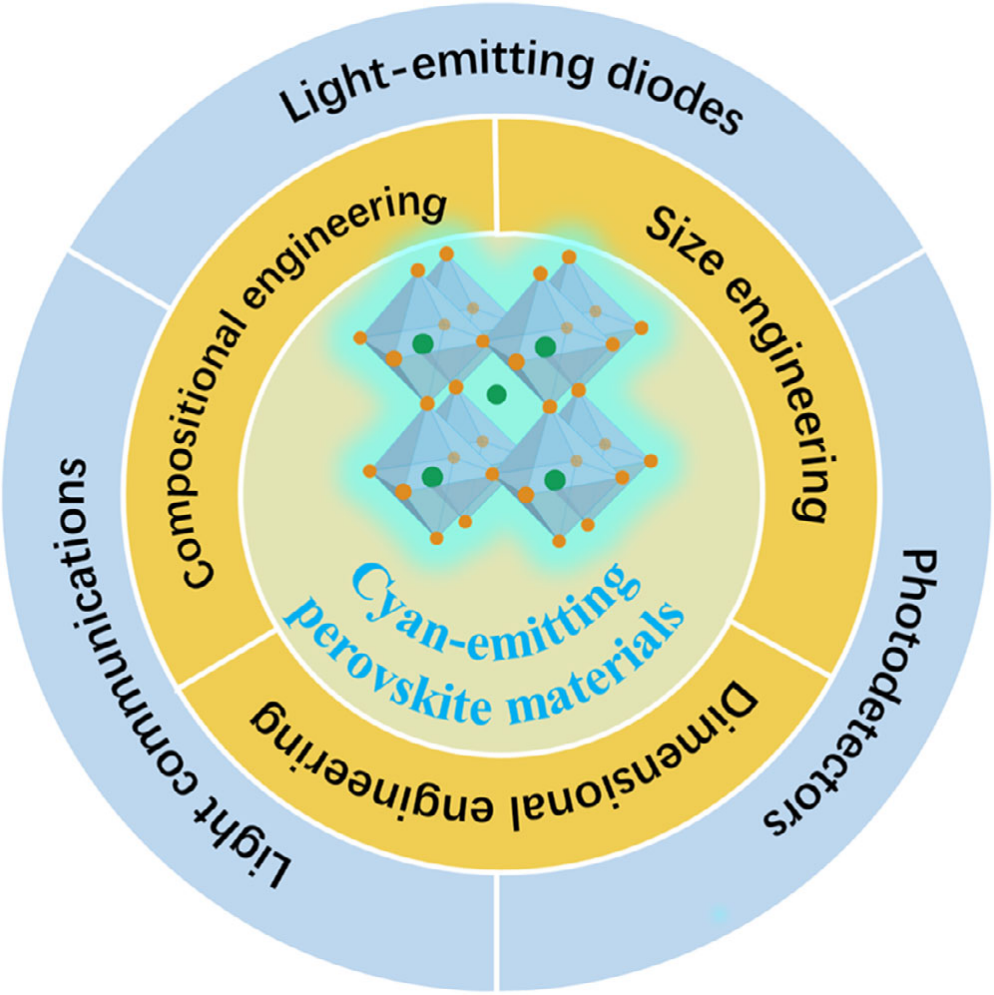
The preparation strategies and potential applications of cyan‐emitting perovskite materials.

The cyan‐emitting perovskites may see lots of applications (**Figure** **2**). On the one hand, the cyan emission could fill the valley between the blue and yellow emission peaks in the conventional white LEDs, resulting in the enhanced color‐rendering index.^[^ ^23^ ^]^ On the other hand, various advantages^[^ ^24^, ^25^ ^]^ (e.g., high quantum yield, ideal bandgap, strong UV light absorption, and high charge carrier mobility with the fast response speed) make these semiconductor materials a potential candidate for photodetector applications.^[^ ^26^ ^]^ Besides, the cyan‐emitting material (480–520 nm) is especially important for underwater communication, because the light attenuation from 430 to 520 nm in the water is much smaller than the other wavelengths.^[^ ^27^ ^]^ Thus, the related devices are highly required, including LEDs, photodetectors, and their signal transmission.^[^ ^28^ ^]^ Therefore, the cyan‐emitting perovskite devices may be integrated into a system for underwater optical communication, such as coastal water and ocean water.^[^ ^24^, ^25^, ^26^, ^29^, ^30^, ^31^, ^32^ ^]^ In addition, the cyan‐wavelength range is an important transition region from green to (deep) blue, whereas most of the preparation strategies of cyan‐emitting perovskites are also possible approaches for the (deep) blue‐emitting perovskites.

In this minireview, we intend to summarize the recent developments of cyan‐emitting perovskite materials and devices. After reviewing the related literature, we catalog the preparation strategies into compositional engineering, dimensional engineering, and size engineering (Figure 2). Compositional engineering, including tuning halogen elements on the X site,^[^ ^33^ ^]^ substituting lead cation on the B site,^[^ ^34^ ^]^ or doping on the A site, is an effective method to tune emission colors to the cyan region.^[^ ^35^ ^]^ The crystal dimension of the above‐mentioned compositional engineering perovskite is still 3D. Another possibility to achieve cyan emission is to replace a small A cation with a larger one partially (e.g., dimethylamine cation) to produce layered perovskite, i.e., 2D/3D perovskites.^[^ ^36^ ^]^ We name this strategy dimensional engineering. Besides the above‐mentioned two methods, it is also possible to realize cyan emission by tuning nanocrystal size, i.e., size engineering.

## 2 Strategies for Preparing Cyan‐Emitting Perovskites

### 2.1 Compositional Engineering for Cyan‐Emitting Perovskite

Compositional engineering is an effective strategy to stabilize the phase and tune the emission colors for halide perovskite. As we know, using mixed halogens at the X site may be one of the most widespread approaches to achieve cyan perovskites, because the bandgap can be tuned simply by controlling the halogen component. However, the halogen ions’ migration often enables undesirable perovskite phase segregation under long‐term radiation and Joule heat. As a result, the electroluminescence (EL) wavelength will undesirably exhibit red shifts during device operation.^[^ ^37^, ^38^, ^39^ ^]^ In contrast, metal doping will not change much of the host crystal structure and basic characteristics, but it can modulate the fundamental optoelectronic properties by introducing heteroatoms. Besides, the ionic substitution (completely or partly) at the A or B site can optimize structure tolerance factor to reduce the lattice strain and endow useful optoelectronic properties by the guest ions as well, including: 1) tune optoelectronic performance, 2) control crystallization process, 3) improve phase stability, etc. The following metallic ions (elements), including rare earth elements (REEs) (e.g., europium, ytterbium, samarium, cerium), main group metal cations (e.g., rubidium, aluminum), and transition metal cations (e.g., manganese), have been successfully doped into perovskite materials for cyan emission without phase separation. In addition, compositional engineering at the B site seems more difficult compared with that of the A and X sites owing to its large formation energy. Therefore, as for preparing cyan‐emitting perovskite, we will focus on metal doping rather than halogen mixing on the X site.

#### 2.1.1 Rare Earth Elements

Due to the unique luminescent properties by D‐band transition in REEs,^[^ ^40^ ^]^ researchers have incorporated them into the perovskite materials and devices to improve the optoelectric properties. Their polyvalent states endow themselves with intriguing redox properties and unique luminous and electromagnetic characteristics.^[^ ^41^, ^42^, ^43^, ^44^, ^45^ ^]^

**Figure 3:**
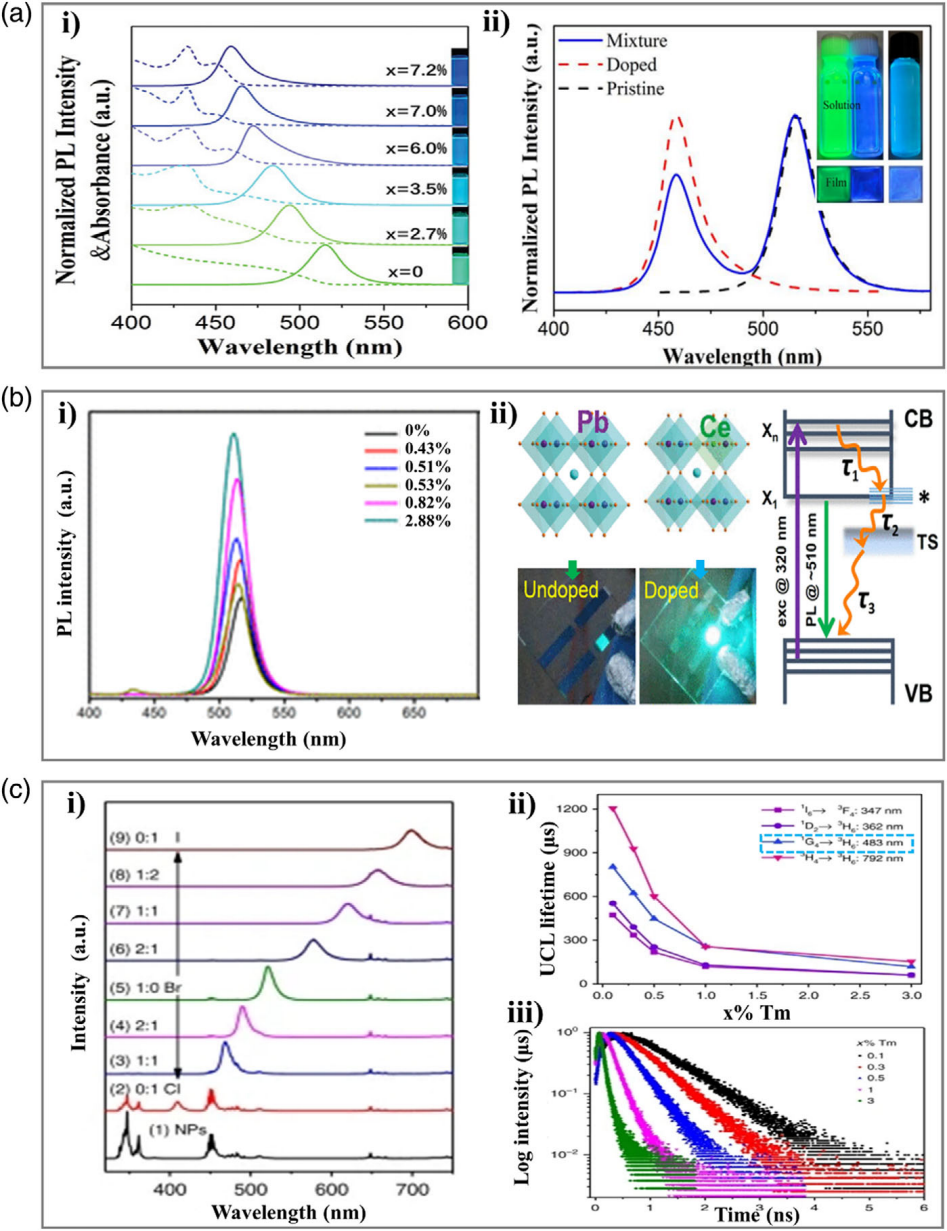
Cyan‐emitting perovskite materials prepared through REE doping. a) Nd^3+^‐doped CsPbBr_3_ perovskite materials. i) PL and absorbance spectra of CsPbBr_3_:*x*Nd^3+^ NC colloidal solutions with different Nd/(Nd + Pb)‐doping ratios: PL emission (solid line, excitation at 365 nm) and UV–visible absorbance spectra (dashed line). The inset shows the corresponding images of luminescent CsPbBr_3_:*x*Nd^3+^ NC colloidal solutions under UV excitation. ii) PL spectra of the mixture of blue CsPbBr_3_:*x*Nd^3+^ (*x* = 7.2%) and green CsPbBr_3_ nanocrystals under excitation of an ultraviolet lamp (365 nm) (the insert is the luminescent perovskite nanocrystals in toluene (top) and encapsulated into PMMA to form a film (bottom) (left: pristine, middle: doped, right: mixed)). Reproduced under the terms of the CC-BY 4.0 license.^[^ ^41^ ^]^ Copyright 2020, The Authors, published by Wiley‐VCH. b) Ce^3+^‐doped CsPbBr_3_ perovskite materials. i) The PL spectra (excitation at 365 nm) of undoped and doped CsPbBr_3_ NCs with different Ce/Pb ratios. ii) The possible structure of undoped and doped perovskite, the photographs of PeLEDs, and the schematic illustration of the involved photophysical processes and mechanisms, where VB, CB, X_1_, and X_*n*_ denote valence band, conduction band, the lowest excitonic state in the CB, and the higher lying excitonic states in the CB, respectively. TS stands for the bandgap trap states, whereas the asterisk depicts the Ce^3+^‐doping‐induced states near the CB band edge. Reproduced with permission.^[^ ^46^ ^]^ Copyright 2018, American Chemical Society. c) Tm^3+^‐doped CsPbX_3_ PeQDs. i) UCL spectra for LiYbF_4_:0.5%Tm^3+^@LiYF_4_ core/shell NPs and the NP‐sensitized CsPbX_3_ PeQDs (NPs: 1 mg mL^−1^, PeQDs: 2 mg mL^−1^) with varying halide compositions under 980 nm continuous‐wave (CW) diode laser excitation at a power density of 50 W cm^−2^. ii) UCL lifetimes of ^1^I_6_, ^1^D_2_, ^1^G_4_, and ^3^H_4_ of Tm^3+^ in LiYbF_4_:x%Tm^3+^@LiYF_4_ core/shell NPs as a function of the Tm^3+^ concentration. iii) UCL decays from the upconverted excitons in NP‐sensitized CsPbBr_3_ PeQDs with varying Tm^3+^ concentration in the NPs. Reproduced under the terms of the CC-BY 4.0 license.^[^ ^47^ ^]^ Copyright 2018, The Authors, published by Springer Nature.

The performance metrics could be highly improved after REE incorporation. This is due to the following reasons: 1) the reduction of (lead or halogen) defects is attributed to the flexible redox properties of REE ion pairs (e.g., Eu^3+^–Eu^2+^),^[^ ^42^ ^]^ 2) the enhancement of the exciton binding energy resulting from the increased electron and hole effective masses, and 3) the increase in oscillator strength ascribed to the shortening of the Pb‐halogen bond. Xie et al.^[^ ^41^ ^]^ reported the preparation of CsPbBr_3_:*x*Nd^3+^ NCs (where *x* is the atomic‐doping ratio *x* = Nd/(Nd + Pb)) by integrating Nd^3+^ into CsPbBr_3_ NCs as B‐site dopants. As shown in **Figure** **3**a‐i, the emission peaks could be finely tuned from blue (459 nm) to cyan (484, 494, and 515 nm, **Table** **1**) with the PLQY values in the range of 75–90% through Nd^3+^ doping. The high PLQY could be ascribed to the reduction of surface zero‐valent lead defects and the increase of exciton binding energy. As the doping ratio increases, the photoluminescence (PL) peak exhibits a continuous blue shift. The theoretical calculation for bulk pristine and doped CsPbBr_3_ reveals that the blue shift is dominated by electronic effects rather than structural doping. The visual cyan emission is achieved by mixing the blue CsPbBr_3_:*x*Nd^3+^ (*x* = 7.2%) NCs and green CsPbBr_3_ NCs, which shows a superimposed emission with two peaks originating independently from the two different types of NCs. Similarly, the dual‐emission perovskite film could be obtained by encapsulating both CsPbBr_3_ NCs and CsPbBr_3_:*x*Nd^3+^ (*x* = 7.2%) NCs in polymethyl methacrylate (PMMA) (Figure 3a‐ii), and then deposited them on the UV‐LED together with a red‐emitting CsPbBr_1.2_I_1.8_/PMMA film. As a result, a white LED with color coordinates optimized at (0.34, 0.33) was obtained, which indicates the decisive role of cyan emission in white LEDs for lighting and displays with better sensory experiences.

**Table 1:** Performance summary of typical cyan‐emitting perovskite materials and devices

Perovskitea)		AF	PL performance			EL performance					Reference
			PLQY [%]	Emission [nm]	FWHM [nm]	EQE [%]	FWHM [nm]	Emission [nm]	Lv [cd m^−2^]	Device structure	
Compositional engineering	Nd^3+^‐doped CsPbBr_3−*x*_ NCs	LED	90(*x *= 7.2%), 75(*x *= 3.5%), 78(*x *= 2.7%),	459(*x *= 7.2%), 484(*x *= 3.5%), 494(*x *= 2.7%),	19(*x *= 7.2%), 24(*x *= 3.5%), 22(*x *= 2.7%),	NA	NA	NA	NA	NA	Xie et al. in 2020^[^ ^41^ ^]^
	Ce^3+^‐doped CsPbBr_3_ NCs	LED	41(undoped), 89(doped),	516(undoped), 510(doped),	NA	1.6(undoped), 4.4(doped),	≈19(undoped), 19(doped)	510(doped)	≈2000(undoped), ≈3500(doped)	ITO/PEDOT:PSS/poly‐TPD/perovskite/TPBi/LiF/Al	Yao et al. in 2018^[^ ^46^ ^]^
	Cs_2_KInCl_6_:5%Sb	PL	93	495	NA	NA	NA	NA	NA	NA	Noculak et al. in 2020^[^ ^50^ ^]^
	PEA_2_(Rb_0.4_Cs_0.6_)_2_Pb_3_Br_10_	LED	84.1	490	NA	1.48	around 30	490	854.3	ITO/PEDOT:PSS/perovskites/TmPyPB/LiF/Al	Jiang et al. in 2019^[^ ^51^ ^]^
	CsPb_0.3_Sn_0.7_Br_3_ QDs	LED	≈20	506	NA	NA	NA	NA	NA	NA	Liu et al. in 2016^[^ ^54^ ^]^
Dimensional engineering	PCPbB film (CsPbBr_3_‐based)	LED	NA	435, 475,	NA	0.015	NA	435, 466, 491,	186	ITO/PEIE‐modified ZnO/perovskite/TFB/MO_*x*_/Al	Cheng et al. in 2017^[^ ^59^ ^]^
	BDADBr‐based MAPbBr_3_	LED	28	494	NA	3.2	NA	494	6800	ITO/PEDOT:PSS/perovskite/TmPyPB/CsF/Al	He et al. in 2020^[^ ^60^ ^]^
	IPA/PEA_2_MA/Cs_*n*‐1_PbnBr_3*n*‐1_	LED	88(477 nm)	477(glass), 477(NiO_*x*_), 478(PVK), 488(PEDOT:PSS)	NA	1.5	NA	481(PVK as HTL)	2480	ITO/HTL/perovskite/TPBi/LiF/Al	Xing et al. in 2018^[^ ^36^ ^]^
	BA_2_DMA_1.6_Cs_2_Pb_3_Br_11.6_ PEA_2_DMA_1.2_Cs_2_Pb_3_Br1_1.2_	LED	63.29, 52.8,	≈487.5 ≈499	NA	2.4(490 nm), 1.58(499 nm)	NA	490, 466,	2825(490 nm), 7760(499 nm),	ITO/PEDOT:PSS/perovskite/TPBi/LiF/Al, ITO/NiO_*x*_/perovskite/TPBi/LiF/Al,	Zeng et al. in 2020^[^ ^62^ ^]^
	CsPbBr_3_:PEACl:YCl_3_	LED	49.7(max)	NA	NA	11	NA	485	9040	ITO/PEDOT:PSS/perovskite/TPBi/LiF/Al	Wang et al. in 2019^[^ ^64^ ^]^
	PEA_2_(EAxCs_1_xPbBr_3_)_2_PbBr_4_	LED	72.85(20%EABr), 72.90(40%EABr), 68.17(60%EABr),	490(20%EABr), 488(40%EABr), 481(60%EABr),	26(20%EABr), 26(40%EABr), 30(60%EABr),	13.3(40%EABr), 12.1(60%EABr), 6.17(80%EABr),	23(40%EABr), 25(60%EABr), 25(80%EABr),	495(40%EABr), 488(60%EABr), 480(80%EABr),	2790(40%EABr), 2191(60%EABr), 83(60%EABr),	ITO/PEDOT:PSS/perovskite/TPBi/LiF/Al	Chu et al. in 2020^[^ ^22^ ^]^
Size engineering	CsPbBr_3_ NCs	PL	52.2(CsPbBr_3_:EDABr_2_:NaBr = 1:0.8:0.03), 81.5(CsPbBr_3_:EDABr_2_:NaBr = 1:1:0), 73(CsPbBr_3_:EDABr_2_:NaBr = 1:1:0.02)	480(CsPbBr_3_:EDABr_2_:NaBr = 1:0.8:0.03), 498(CsPbBr_3_:EDABr_2_:NaBr = 1:0.4:0.03), 498(CsPbBr_3_:EDABr_2_:NaBr = 1:1:0), ≈495(CsPbBr_3_:EDABr_2_:NaBr = 1:1:0.02)	25(CsPbBr_3_:EDABr_2_:NaBr = 1:0.8:0.03), 26(CsPbBr_3_:EDABr_2_:NaBr = 1:0.4:0.03), 22(CsPbBr_3_:EDABr_2_:NaBr = 1:1:0)	NA	NA	NA	NA	NA	Worku et al. in 2020^[^ ^67^ ^]^
	Crown‐treated PEA‐based CsPbBr_3_	LED	≈9(100%PEABr), 70±8(40%PEABr‐Crown)	528(0%PEABr), 507(100%PEABr)	NA	NA	15.5(40%PEABr‐Crown)	510(60%PEABr‐Crown)	7000(40%PEABr), 19540(40%PEABr‐Crown)	ITO/Poly‐TPD/perovskite/TPBi/LiF/Al	Ban et al. in 2018^[^ ^71^ ^]^

a) Photoluminescence (PL); electroluminescence (EL); application filed (AF); photoluminescence quantum yield (PLQY); full width at half maxima (FWHM); external quantum efficiency (EQE); luminance (Lv); reference (Ref.); nanocrystals (NCs); light‐emitting diode (LED); data was estimated from the original figure (≈); not applicable (NA); indium tin oxide (ITO); poly(3,4‐ethylenedioxythiophene):poly(styrene sulfonate) (PEDOT:PSS); poly[*N*,*N*′‐bis(4‐butylphenyl)‐*N*,*N*′bisphenylbenzidine] (poly‐TPD); poly(9‐vinlycarbazole) (PVK); C_6_H_5_C_4_H_8_NH_3_Br; “4‐PBABr”‐modified CsPbBr_3_ (PCPbB); polyethylenimine ethoxylated (PEIE); poly(9,9‐dioctyl‐fluorene‐co‐N‐(4‐butylphenyl)diphenylamine) (TFB); molybdenum oxide (MO_*x*_); 1,4‐diaminobutane hydrobromide (BDADBr); 1,3,5tri(m‐pyrid‐3‐yl‐phenyl)benzene (TmPyPB); iso‐propylammonium (IPA); 2‐phenethylamine (PEA); n‐butylammonium (BA); dimethylammonium (DMA); C_2_N_2_H_10_Br_2_ (EDABr_2_); and 1,4,7,10,13,16‐hexaoxacyclooctadecane (Crown).

REEs’ large stokes shift has great potential to obtain cyan‐emitting perovskite materials with satisfactory optical/electric properties. In 2018, Yao et al.^[^ ^46^ ^]^ proposed a novel strategy to enhance the PL/EL efficiency of CsPbBr_3_ NCs via doping cerium ion (Table 1). With the increasing doping amount of Ce^3+^ in perovskite NCs to 2.88% (atomic percentage of Ce/Pb), the peak PL reached up to the strongest intensity (Figure 3b‐i). The corresponding PLQY of CsPbBr_3_ NCs reached up to 89%, by a factor of 2 in comparison with the undoped ones (PLQY ≈41%). Noticeably, it is easy to find that there exists a blue shift (6 nm, from 516 to 510 nm, pale cyan to deep cyan) of the PL emission peak of perovskites with increasing the doping amount. However, the authors concluded that the quantum confinement effect caused by the size variation was not responsible for the PL spectral shift here. Instead, it plays the dominant role in electronic doping by REEs. In other words, the CB filling by the donated electrons (Moss–Burstein effect) can result in a blue shift in the spectra of heavily doped n‐type semiconductors (Figure 3b‐ii). The following PeLED device fabricated using the REEs‐doped CsPbBr_3_ NCs, as the active emitting layers exhibited a great improvement of EQE from 1.6 to 4.4%.

REEs doping can prolong the luminescent lifetimes of the excitons. Zheng et al.^[^ ^47^ ^]^ synthesized various LiYbF_4_:*x*% (*x *= 0.1–3) Tm@LiYF_4_‐sensitized CsPbX_3_ (X = Cl, Br, I) with long upconversion luminescence (UCL) lifetimes. They first obtained full‐color emissive LiYbF_4_:0.5%Tm^3+^@LiYF_4_ core/shell nanoparticles (NP)‐sensitized CsPbX_3_ perovskite quantum dots (PeQDs) with varying halide compositions (Figure 3c‐i). As shown in Figure 3c‐ii, through lifetime tuning of Tm^3+^ in the perovskite NPs, the UCL lifetimes of perovskite QDs were then modulated. By controlling the Tm^3+^ concentration from 3 to 0.1 mol% in the NPs, the lifetimes of ^1^I_6_, ^1^D_2_, ^1^G_4_, and ^3^H_4_ of Tm^3+^ increased from 60, 61, 119, and 154 μs to 473, 553, 803, and 1205 μs, respectively. Accordingly, the UCL lifetimes of CsPbCl_3_, CsPbBr_3_, and CsPbI_3_ PeQDs sensitized by the NPs were tuned from 61, 81, and 80 μs to 494, 794, and 1053 μs, respectively (Figure 3c‐iii).

Therefore, we can find that the REEs are beneficial to improve cyan‐emitting perovskite materials’ optical performances, including to reduce defects, improve PLQY, tune emission wavelengths, and prolong luminescent lifetime. However, for most REEs‐doping perovskite materials, one of the major challenges in these perovskite materials may be stability. This is mainly because of the ionic size mismatch between most REEs and Pb or Cs. It is noted that the radius of Eu^2+^ ion is very close to that of Pb^2+^ ion. Undoubtedly, the REEs are far less abundant than nonrare earth metals, and it is, thus, important to explore the roles of other metal ions in perovskites with cyan emission.

#### 2.1.2 Other Metal Elements

Encouragingly, there are many reports on introducing other metal elements (ions) into perovskite materials. Similar to the introduction of REEs, the realizability of adopting other metal elements (ions) is ensured, because the metals at the B site (in ABX_3_ form) play an important role in determining the whole electronic structure, optical, and photoelectric properties. In addition, most nonrare earth metals (and their mineral resources) own huge storage capacities globally. Therefore, to effectively and controllably dope appropriate nonrare earth metal ions into perovskite is merited to be performed.

Usually, metal ions (e.g., Mn^2+^) prefer to be absorbed onto the surface of perovskite QDs rather than to be incorporated in the QDs, indicating that the energy transfer from the exciton to metal ions excited states is not efficient due to the weak coupling of the exciton to the remote metal ions. Mn ion doping has been proved to be a contributory approach to prepare CsPbCl_3_ QDs with orange emission around 600 nm.^[^ ^48^ ^]^ Adopting aluminum (Al) ions into perovskite materials caused a blue shift in the PL emission wavelengths. For example, Liu et al.^[^ ^49^ ^]^ reported that Al^3+^ doping could endow CsPbBr_3_ perovskite NCs a blue emission (456 nm), whereas the pristine CsPbBr_3_ shows a cyan PL emission wavelength (515 nm). This is because the Al^3+^ doping brings in a new energy level, which is from the hybridization of the Al s‐orbitals, the Br p‐orbitals, and the Pb p‐orbitals.

**Figure 4:**
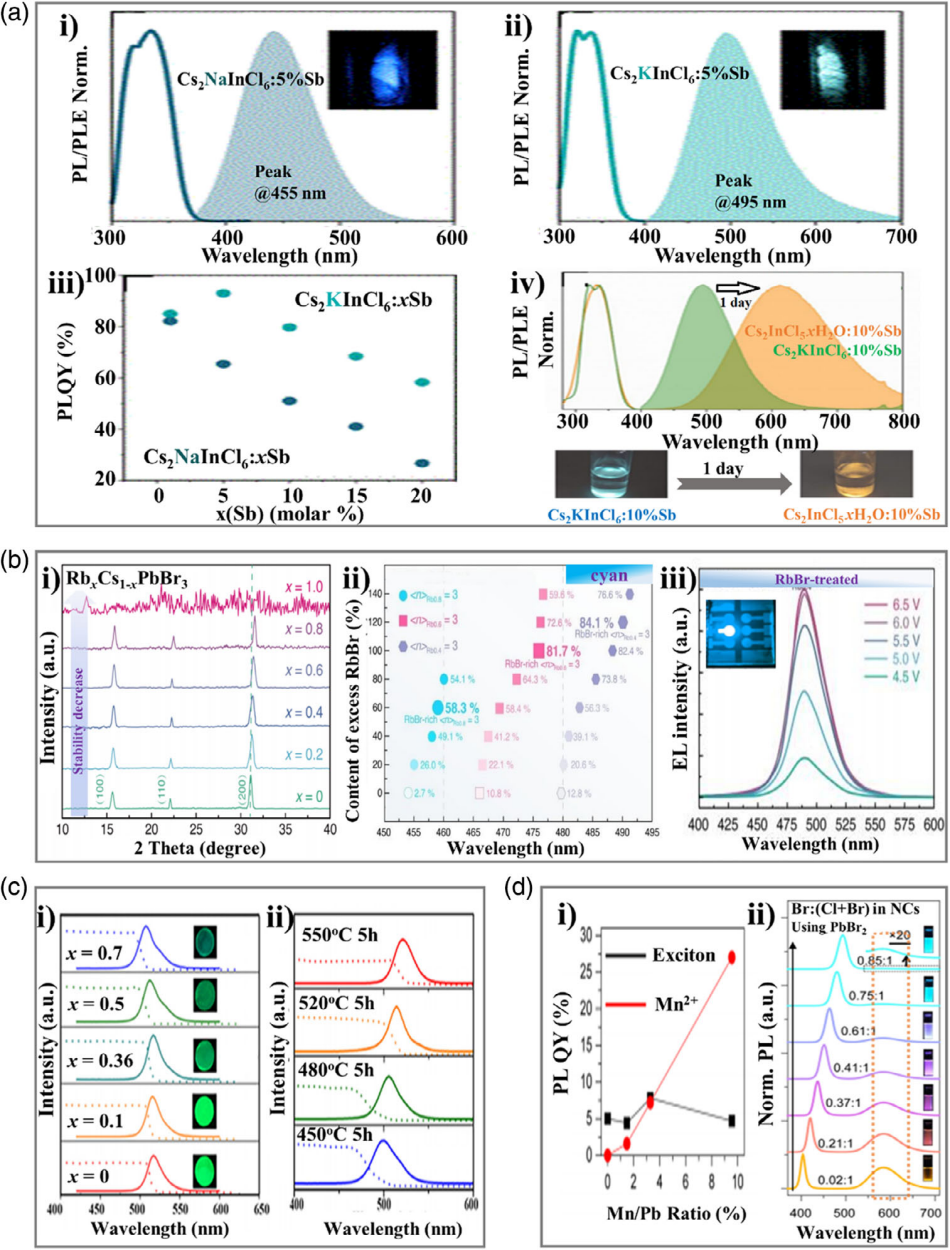
Cyan‐emitting perovskite materials prepared through other metal ions doping. a) Trivalent antimony (Sb(III))‐doping Cs_2_NaInCl_6_:5%Sb and Cs_2_KInCl_6_:5%Sb. i,ii) Typical PL (under 320 nm UV excitation) and photoluminescence excitation (PLE) of Cs_2_NaInCl_6_:5%Sb and Cs_2_KInCl_6_:5%Sb, respectively. iii) PLQY dependence on the concentration of the Sb^3+^ dopant. iv) PL/PLE of Cs_2_KInCl_6_:10%Sb measured before and after conversion to Cs_2_InCl_5_ *x*H_2_O:10%Sb. Images at the bottom are the powder emission under UV excitation immediately after precipitation and after 1 day. Reproduced with permission.^[^ ^50^ ^]^ Copyright 2020, American Chemical Society. b) Rb‐doped CsPbBr_3_‐based perovskite. i) X‐ray diffraction (XRD) patterns of 3D Rb_*x*_Cs_1‐*x*_PbBr_3_ perovskites. ii) PL emission wavelengths and corresponding PLQYs of the quasi‐2D perovskites. iii) EL spectra of RbBr‐treated perovskite‐based PeLEDs at different voltage biases (the insert is the photograph of PeLED). Reproduced under the terms of the CC-BY 4.0 license.^[^ ^51^ ^]^ Copyright 2019, The Authors, published by Springer Nature. c) Sn‐doped CsPbBr_3_ QDs. i) Intensities of the absorption and emission spectra of CsPb_1‐*x*_Sn_*x*_Br_3_ (*x* = 0, 0.1, 0.36, 0.5, and 0.7) at 550 °C, respectively. Inset: CsPb_1−*x*_Sn_*x*_Br_3_ glasses under the irradiation of a 365 nm UV lamp. ii) UV–vis absorption and PL emission of CsPb_0.64_Sn_0.36_Br_3_ at different heat‐treatment temperatures. Reproduced with permission.^[^ ^52^ ^]^ Copyright 2019, Elsevier. d) Mn^2+^‐doped CsPbX_3_ nanocrystals (where X = Cl, Br, or I). i) The effect of dopant concentration onto the PLQY of Mn:CsPbCl_3_ NC. ii) PL spectra of NCs taken during progressive Br^−^ anion exchange using PbBr_2_, starting with CsPbCl_3_ NCs. Spectra are normalized at the peak of the band‐edge (shorter wavelength) emission feature. Reproduced with permission.^[^ ^54^ ^]^ Copyright 2016, American Chemical Society.

Apart from the role in tuning the bandgap of perovskite materials, we think the metal ions have the potential to be used as an emissive unit due to their intrinsic upconversion properties. According to a very recent work reported by Noculak et al.,^[^ ^50^ ^]^ trivalent antimony (Sb^3+^) was incorporated into perovskite as a bright emissive center for lighting. They first prepared a series of perovskites using Sb^3+^ as a dopant in a solution‐grown metal halide double perovskite (DP) matrix, namely, Cs_2_MInCl_6_:*x*Sb (M = Na, K, *x* = 0–100%). Moreover, the PL emission is situated in a wide range (380–700 nm). As shown in **Figure** **4**a‐i,ii, both blue and cyan emissions were obtained from Cs_2_NaInCl_6_:5%Sb (445 nm) and Cs_2_KInCl_6_:5%Sb (495 nm), respectively. The amount of Sb^3+^ dopant strongly influences the PLQY values (Figure 4a‐iii). The champion PLQYs (82% and 93%) were obtained for the Cs_2_NaInCl_6_:1%Sb and Cs_2_KInCl_6_:5%Sb (*λ*_excitation_ = 320 nm), respectively (Table 1). The longest PL lifetime can be achieved by adjusting the doping concentration, and the maximal lifetime of cyan‐emitting perovskite was obtained when the doping concentration was 5%. Furthermore, the Sb^3+^ dopant concentrations with differences have no obvious negative effects on PL stability (peak PL position). However, the performance of its storage stability is not very well (Figure 4a‐iv), due to the destruction of water.

To incorporate rubidium ion (Rb^+^) into perovskite is another approach to tune the emission wavelength, which can be approved by the work conducted by Jiang et al. in 2019.^[^ ^51^ ^]^ The authors doped Rb^+^ (RbBr) into the A site of CsPbBr_3_ first, and then, the 3D Rb–Cs alloyed perovskites (Rb_*x*_Cs_1‐*x*_PbBr_3_) with a tunable bandgap (2.31–2.60 eV, 0 ≤ *x* ≤ 0.8) were obtained. Among the whole Rb–Cs alloyed perovskites, the Rb_0.6_Cs_0.4_PbBr_3_ is the only stabilized cyan emitter (499 nm), with an unsatisfactory PLQY (0.7%) with a short PL lifetime (around 0.4 ns). The stability of Rb_*x*_Cs_1‐*x*_PbBr_3_ shows a downward trend as the Rb^+^ ratio increases, which ascribes to the increased octahedral distortion caused by continuous Rb^+^ substitution (Figure 4b‐i). Subsequently, the authors fabricated PEA_2_(Rb_*x*_Cs_1‐*x*_)_2_Pb_3_Br_10_ (0 ≤ *x* ≤ 1) perovskites with different Rb^+^ contents to improve the PLQY as well as stability. As expected, the PLQY was improved (e.g., PLQY = 11%, PEA_2_(Rb_0.6_Cs_0.4_)_2_Pb_3_Br_10_). However, the PL wavelength shifted to a blue region (466 nm) due to the strong dimension contribution from the phenylethylammonium bromide (PEABr) additive. Excess RbBr was further incorporated for improving the PLQY as well as tune the emission wavelength. For example, the excess RbBr‐treated PEA_2_(Rb_0.8_Cs_0.2_)_2_Pb_3_Br_10_ and PEA_2_(Rb_0.4_Cs_0.6_)_2_Pb_3_Br_10_ exhibit the PLQYs of 58.3% and 84.1%, with an emission at 458 nm (blue) and 490 nm (cyan), respectively (Figure 4b‐ii and Table 1). The cyan‐emitting PeLEDs exhibited a peak EQE of 1.48% (T50 lifetime of 18.7 min), with good EL stability (Figure 4b‐iii).

Besides the poor humidity stability and crystal stability, the phase instability of halide‐based perovskites is another issue to be solved. Fortunately, compositional engineering achieved through doping on A or B sites has been proved as an alternative approach to tune the emission of perovskite light‐emitting materials without phase separation. In 2019, Liu et al.^[^ ^52^ ^]^ synthesized tin(Sn)‐doped CsPbBr_3_ perovskite nanocrystals in glasses by traditional melt‐quenching. The positions of the peak PL of perovskites were successfully tuned from pale cyan (518 nm, *x* = 0) to a cyan range (506 nm, *x* = 0.7), as the amounts of Sn dopant increase (Figure 4c‐i). The tunable PL shift is attributed to the contraction of the lattice with Sn doping. As shown in Figure 4c‐ii), the PL emission wavelengths were also tuned by changing the heat‐treatment temperature, which can be explained by the quantum size effect of perovskite QDs. Specifically, the bandgap of Sn‐incorporated CsPbBr_3_ QDs materials gradually decreases, as the heat‐treatment temperature increases. Besides, the Sn‐doped CsPbBr_3_ QDs exhibited excellent air stability and thermal stability due to the protection from borosilicate glasses (B_2_O_3_–SiO_2_–ZnO [BSZ]). Significantly, apart from the effective prevention of phase separation, this work also provides us another direction to ensure the stability of perovskite materials (e.g., to introduce effective coating materials and technology as a protection layer).

Moreover, the adoption of trivalent indium (In^3+^) cations has also been proved beneficial to overcome the phase instability of perovskite. In 2019, Wu et al.^[^ ^53^ ^]^ adopted In^3+^ for effective substitution at the B site of CsSnCl_3_ perovskite. The optimized perovskite (CsSn_0.9_In_0.067_Cl_3_) shows a PL peak located in the cyan region (484 nm). Excitingly, the phase stability of this perovskite material was significantly improved when compared with the pristine CsSnCl_3_. Disappointingly, the PLQY of CsSn_0.9_In_0.067_Cl_3_ is below the detection limit of the apparatus. In our opinion, the further enhancement of the overall PL/EL performance of these perovskites is encouraging. The passivation engineering, additive engineering, and other methods may be possible considerations.

As previously described, divalent Mn ions own a stable emission around 586 nm due to Mn^2+^ intrinsic d–d emission, which is independent of dopant concentration. Therefore, Mn doping for overall cyan emitting may enable a new kind of perovskite luminescent material. In 2016, Liu et al.^[^ ^54^ ^]^ developed an effective approach for incorporating Mn ions into CsPbX_3_ nanocrystals (where X = Cl, Br, or I) (Table 1). First, the doping amount has obvious effects on the PLQY values. As shown in Figure 4d‐i, the PLQY of Mn^2+^ transition increased proportionately with the doping level, up to a maximum of 27% for NCs (containing 9.6% Mn relative to Pb). For example, as shown in Figure 4d‐ii, the obtained Mn:CsPbCl_3‐*x*_Br_*x*_ NCs exhibited a wide PL emission range from overall orange to the cyan region. It is noteworthy that the emission contributions (marked with dotted frame) from Mn always existed though the ratio of Br were continuously increased, indicating that the strong Mn emission can be retained. Similarly, a series of dual‐emissive Mn‐doped CsPbCl_3‐*x*_Br_*x*_ NCs were achieved with an indispensable Mn emission contribution.

It shows that the proper metal‐doping strategy indeed provides us with effective approaches to endow target perovskites with desirable performances. Doping metal ions into perovskite materials is beneficial to tune the bandgap, improve light absorption ability, suppress trap states, and adjust the energy level.^[^ ^55^ ^]^ The incorporation of metal ions into the perovskite layer has an important effect on controlling the majority of the carrier type of perovskite materials.^[^ ^56^ ^]^

As for cyan‐emitting perovskites prepared through nonmetal doping, it is usually achieved through organic additive (e.g., dimethylammonium hybromide), followed by a reduced dimension. Therefore, we will discuss nonmetal doping as dimensional engineering in the next section.

### 2.2 Dimensional Engineering for Cyan‐Emitting Perovskite

If the small A^+^ is replaced by a much larger organic ammonium cation, the 3D perovskite will change to the 2D layered structure due to steric effects. The general formula of 2D perovskite is (L)_2_(S)_*n*‐1_B_*n*_X_3*n*+1_, where L and S are long/short‐chain ammonium cations, B is a divalent metal ion, and X is a halide anion. Due to the phase competition, it usually forms 2D/3D composition. The 2D/3D perovskite will be self‐organized into multiple quantum wells, and it works as an energy funnel to boost the luminescent performance of low energy states. Hence, the A cation plays a crucial role in determining the perovskite structure and dimensionality. Following this line, many works through adding ammonium cations have been conducted to obtain luminescent perovskites with low dimensions, mainly at A site.

**Figure 5:**
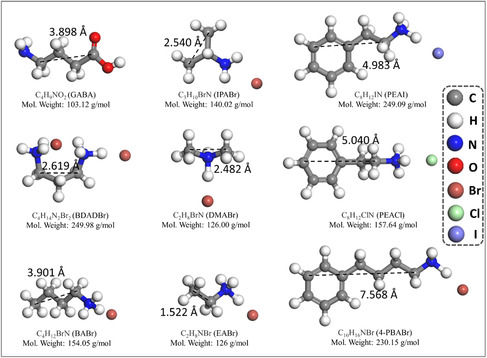
Typical ammonium additives for preparing cyan‐emitting perovskite materials through dimensional engineering. In this figure, the gray balls are C atoms, the blue balls are N atoms, the white balls are H atoms, the red balls are O atoms, the dark red balls are Br atoms, the green balls are Cl atoms, and the purple balls are I atoms in these structural representations. While, the abbreviations of GABA, IPABr, PEAI, BDADBr, DMABr, PEACl, BABr, EABr, and 4‐PBABr represent γ‐aminobutyric acid, isopropylamine hydrobromide, phenethylammonium iodide, 1,4‐diaminobutane hydrobromide, dimethylammonium hybromide, phenylethylammonium chloride, butylamine hydrobromide, ethylammonium bromide, and 4‐phenylbutylamine hydrobromide, respectively.

**Figure 6:**
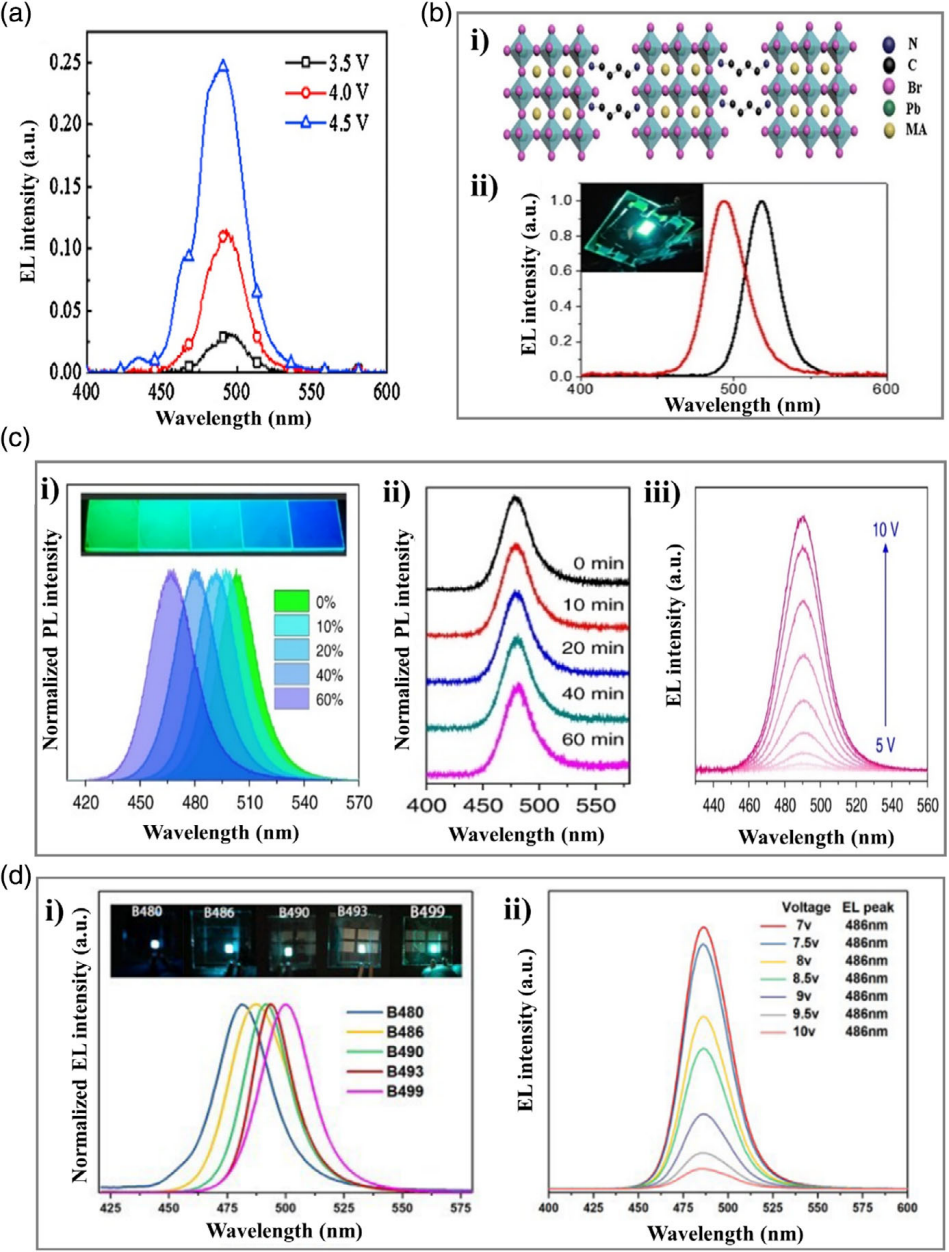
Cyan‐emitting perovskite materials and devices prepared through dimensional engineering. a) EL spectra of the 4‐PBABr‐based quasi‐2D perovskite. Reproduced with permission.^[^ ^59^ ^]^ Copyright 2017, Elsevier. b) BDADBr‐based quasi‐2D perovskite. i) Semantic configurations of BDADBr‐based quasi‐2D perovskite. ii) EL spectra of the BDADBr‐based quasi‐2D perovskite (black line represents MABr:BDADBr = 8:1, and red line represents MABr:BDADBr = 2:1, molar ratio). Reproduced with permission.^[^ ^60^ ^]^ Copyright 2020, Springer. c) PEA‐based perovskite with IPABr. i) PL spectra of PEA_2_A_1.5_Pb_2.5_Br_8.5_ perovskite with 0‐60% IPABr additive (the inset is a photograph of the corresponding films under 365 nm UV irradiation). ii) PL spectra stability of perovskite under continuous laser radiation (325 nm, 7 W cm^−2^) for different exposure times. iii) EL spectra of PeLEDs operating under different voltage. Reproduced under the terms of the CC-BY 4.0 license.^[^ ^36^ ^]^ Copyright 2018, The Authors, published by Springer Nature. d) Quasi‐2D perovskite by mixing ammonium ligands. i) EL spectra and photographs of PeLED (B480 represents DMABr:BABr = 1, molar ratio, PEDOT:PSS as HTL, Annealed@65 °C, B460 represents DMABr:BABr = 1, molar ratio, PEDOT:PSS as HTL, Annealed@70 °C, B490 represents DMABr:BABr = 0.8, molar ratio, PEDOT:PSS as HTL, Annealed@70 °C, B493 represents DMABr:PEABr = 0.6, molar ratio, NiO_*x*_ as HTL, Annealed@70 °C, and B499 represents DMABr:PEABr = 0.6, molar ratio, PEDOT:PSS as HTL, Annealed@70 °C). ii) EL intensity of PeLEDs under different voltages. Reproduced with permission.^[^ ^62^ ^]^ Copyright 2020, The Royal Society of Chemistry.

First, various ammonium additives with different chain lengths could be used for reducing the dimension of perovskite to realize cyan emission (**Figure** **5**). For example, the butylammonium interlayers^[^ ^57^ ^]^ were used to obtain Ruddlesden–Popper inorganic mixed halide perovskites, and the following PeLEDs show a cyan emission (EQE = 6.2%, emission at 487 nm). The GABA^[^ ^58^ ^]^ was proved to be effective in limiting the formation of larger *n* values of perovskite materials and then stabilizing phase. Cheng et al.^[^ ^59^ ^]^ adopted C_6_H_5_C_4_H_8_NH_3_Br (4‐PBABr) into the precursor to reduce the dimension of cesium (Cs)‐based perovskite and obtained 2D perovskite (4‐PBABr:CsBr:PbBr_2_=2:1:1, molar ratio) with good film coverage. The corresponding PeLEDs exhibited a sky‐blue emission (**Figure** **6**a) with a dominant EL peak at 491 nm and a maximum EQE of 0.015% at the brightness of 186 cd m^−2^ (Table 1). Adopting a similar approach, He et al.^[^ ^60^ ^]^ added cross‐linked BDADBr into perovskite precursor to tune the emission wavelength of MAPbBr_3_ (MABr:PbBr_2_ = 1.2:1, molar ratio) perovskite materials (see Figure 6b‐i). The PL emission shift from green (3D MAPbBr_3_) to a cyan (sky‐blue, 494 nm) light region due to the formation of the 2D/3D quantum well and the PLQY was increased up to 28%. The enhancement of PLQY might be dominated by the carrier density, whereas the basic carrier decay physics is determined by the structural dimension of the perovskite, regardless of the composition.^[^ ^61^ ^]^ Hence, the formation of the 2D/3D quantum well should be beneficial to speed up the corresponding recombination process for the following high‐efficiency LEDs. As a result, the optimized perovskite was further used to fabricate LED device with polyethylene for passivating defects, and the as‐fabricated PeLED showed an EL maximum at 494 nm and a champion EQE of 3.2% (Figure 6b‐ii and Table 1).

Although 2D/3D perovskite can improve the luminescent properties, PeLED emission requires large driving voltages due to the insulating nature of large organic cations. Xing et al.^[^ ^36^ ^]^ reported color‐stable sky‐blue (cyan) PeLEDs achieved by enhancing the phase stability of quasi‐2D perovskite thin films. First, the PL peak of perovskite shifted progressively from 497 to 467 nm, as the IPABr/Pb ratio (IPABr represents the isopropylamine hydrobromide) increases from 10 to 60% (Figure 6c‐i), whereas the pristine PEA_2_A_1.5_Pb_2.5_Br_8.5_ (A = MA and Cs) perovskite emitted at 504 nm. The highest PLQY of 73% (emission at 480 nm) was obtained with an IPABr ratio of 40%. The perovskite film with 40% IPABr exhibited good PL stability under continuous 325 nm laser radiation. As shown in Figure 6c‐ii, the PL maximum is unchanged even after irradiation for 60 min, which can be attributed to the phase stability of perovskite. The corresponding PeLEDs exhibit stable sky‐blue emission under high operation voltages (Figure 6c‐iii), which is mainly attributed to the branched molecular structure of IPABr (see Figure 5). The EL spectra were further tuned using different hole transport layer (HTL) materials, including PEDOT:PSS, PVK, and NiO_*x*_. The corresponding EL peaks were located at 490 nm (PEDOT:PSS), 481 nm (PVK), and 477 nm (NiO_*x*_), respectively. Finally, a maximum luminance of 2480 cd m^−2^ at 490 nm was achieved (Table 1).

In 2020, Zeng et al.^[^ ^62^ ^]^ provided another efficient method to obtain quasi‐2D sky‐blue PeLEDs by adopting a mixed ammonium spacer to reduce the perovskite dimension. Unlike most previous reports, Zeng and co‐workers used mixed ammonium ligands (DMABr+BABr, DMABr+PEABr) rather than single organic ammonium. The molecule size and charge on amino‐groups of the various organic ammoniums were different. As expected, the authors found that the molecule size and charge on amino‐groups of the co‐ligand hold the keys to construct an effective mixed‐ligands system. Dimethylammonium (DMA) was selected as an ideal co‐ligand in both BA‐ and PEA‐based quasi‐2D perovskites due to its proper molecular size. As expected, DMA could help to confine the diameter of perovskite nanocrystals (NCs) and improve their dispersity in organic ligands. Besides, DMA is prone to disperse among PEA or BA molecules to cap the perovskite synergistically. The phase of *n *= 1 caused by excess co‐ligands could be effectively avoided benefiting from the relatively weak coordination ability of DMA because of the relatively small positive charge on amino‐groups. The optimized PeLEDs based on BA_2_DMA_1.6_Cs_2_Pb_3_Br_11.6_ and PEA_2_DMA_1.2_Cs_2_Pb_3_Br_11.2_ afford effective cyan emission at 490 and 499 nm, respectively (Figure 6d‐i), with a record maximum luminance of 2825 and 7760 cd m^−2^, respectively (Table 1). Moreover, as shown in Figure 6d‐ii, the PeLEDs also display high spectra stability at high voltage, which may be the result of the branched DMA‐based additive. We think that the positive effect of branched DMABr, in this case, is similar to the last example of the IPABr additive partly.

**Figure 7:**
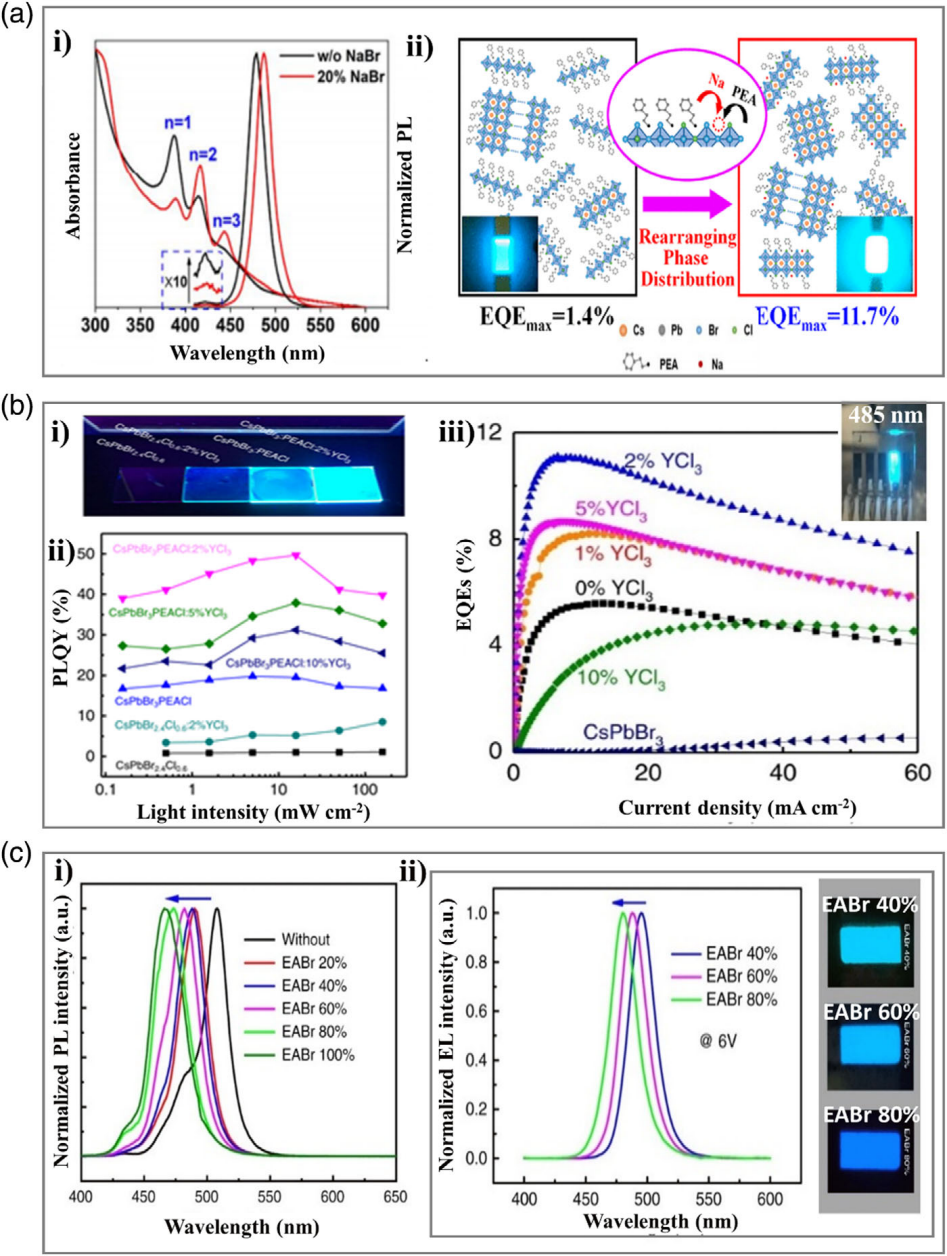
Cyan‐emitting perovskite materials prepared through dimensional engineering combined with other methods. a) Rearrangement of phase distribution in quasi‐2D perovskites. i) Absorption and PL spectra. ii) Schematic illustration of the rearrangement of phase distribution by adding Na^+^ in quasi‐2D perovskites. Reproduced with permission.^[^ ^63^ ^]^ Copyright 2020, American Chemical Society. b) A 2D and 3D perovskite through dimensional engineering and REEs doping. i) A photograph of the films under a UV lamp. ii) Power‐dependent PLQYs of the perovskite films with different compositions. iii) The EQE–current density curves of PeLED, and the insert photograph is the fabricated PeLED showing cyan EL emission. Reproduced under the terms of the CC-BY 4.0 license.^[^ ^64^ ^]^ Copyright 2019, The Authors, published by Springer Nature. c) Ethylammonium incorporated quasi‐2D perovskite. i) Normalized PL spectra. ii) Normalized EL spectra of PeLEDs (left), and EL images of the PeLEDs (right). Reproduced under the terms of the CC-BY 4.0 license.^[^ ^22^ ^]^ Copyright 2020, The Authors, published by Springer Nature.

The combination of dimensional engineering and other methods (e.g., metal doping) is proved to be another effective strategy for preparing cyan‐emitting perovskites. For example, Pang et al.^[^ ^63^ ^]^ introduced sodium ions (Na^+^) into the quasi‐2D perovskites with phenylethylammonium as the organic spacer and cesium lead halide as the inorganic framework. The inclusion of the Na^+^ was found to significantly increase the formation of other small‐*n* phases for efficient exciton energy transfer (**Figure** **7**a‐i). As a result (Figure 7a‐ii), a maximum EQE of 11.7% was achieved in the cyan PeLED (emission peak at 488 nm). Similarly, REEs doping into low dimensional perovskite was another approach to achieve cyan‐emitting PeLED. For example, in 2019, Wang et al.^[^ ^64^ ^]^ mixed PEACl into cesium lead halide perovskites that yield a mixture of 2D/3D perovskites, which enhances PLQY increased from 1.1% to 19.8% (Figure 7b‐i,ii). Adding REEs (Y^3+^) into the mixture further enhances PLQY to 49.7%. As for the cyan‐emitting PeLED, the device with PEACl:CsPbBr_3_ (1:1) has an EQE of 5.6% and a maximum brightness of 5183 cd m^−2^, whereas adding 2% YCl_3_ in the perovskite film significantly increases EQE and maximum luminance to 11.0% and 9040 cd m^−2^, respectively (Figure 7b‐iii and Table 1). The excellent performance is because of the reduction of dimension resulting from PEACl additive as well as the increase in the bandgap of perovskite grain shell through REEs doping.

Besides, passivation technology can also be performed to improve the overall performance of 2D/3D perovskite. For example, in 2020, Chu et al.^[^ ^22^ ^]^ incorporated a large cation (CH_3_CH_2_NH_2_^+^, EA^+^) with 2D CsPbBr_3_‐based perovskite to achieve a stable cyan‐emitting PL and EL performance (Figure 7c‐i). As a result (Figure 7c‐ii,iii and Table 1), the PL emissions were tuned to the cyan region by controlling the amount of EABr (e.g., 40% EABr, PL at 488 nm, PLQY = 72.9%), whereas the champion EQE of 12.1% (EL located at 488 nm) was demonstrated with 60% EABr. In this work, the perovskite materials enabled excellent performances because: 1) the PEABr (a long alkyl chain phenylethylammonium bromide) was introduced in the perovskites to form 2D phases, resulting in stable performance and 2) the EABr doping leads to effective suppression of nonradiative recombination as well as tune the emission wavelength and optical bandgap.

From the above‐mentioned examples, we conclude that: 1) suitable spacer additives are important for cyan‐emitting perovskites, because the chemical structure of the linking ammonium spacer has great influences on the performance of perovskite materials and devices. 2) The synergistic effect of combining different strategies is essential for improving the performance of perovskite materials and devices.

### 2.3 Size Engineering for Cyan‐Emitting Perovskite

**Figure 8:**
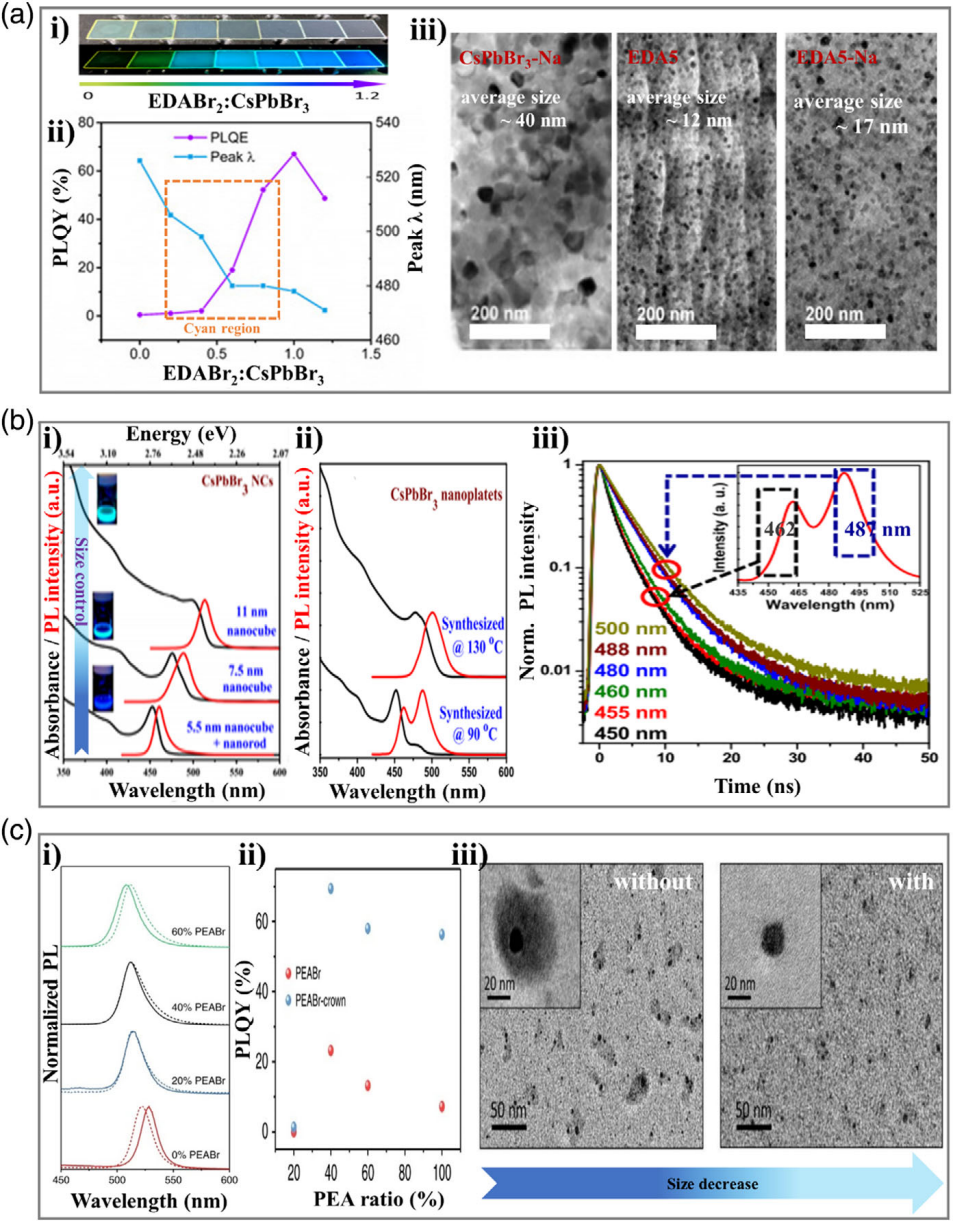
Cyan‐emitting perovskite materials prepared through size engineering. a) Hollow CsPbBr_3_ NCs containing 3 mol% NaBr and varying concentrations of EDABr_2_ additive. i) Thin films with various concentrations of EDABr_2_ under ambient light (top) and ultraviolet (UV) irradiation (bottom). ii) PLQY and emission peak wavelength of thin films with EDABr_2_ molar ratio varying from 0 to 1.2 to the perovskite precursors. iii) Transmission electron microscopy (TEM) images of CsPbBr_3_‐Na, EDA5, and EDA5‐Na thin films. Reproduced with permission.^[^ ^67^ ^]^ Copyright 2020, American Association for the Advancement of Science. b) CsPbBr_3_ perovskite nanocube and nanoplatelets. i) UV–visible absorption and PL spectra of CsPbBr_3_ NCs prepared at 190 °C (11 nm nanocube), 140 °C, first fraction of size selective precipitation (7.5 nm nanocube), and 140 °C second fraction of size selective precipitation (5.5 nm nanocubes + nanorods). ii) UV–visible absorption and PL spectra CsPbBr_3_ NCs prepared at 130 °C and 90 °C, mainly containing nanoplatelets. iii) PL decay dynamics of CsPbBr_3_ NCs prepared at 90 °C. Inset is the corresponding PL spectrum. Arrows of different colors indicate the emission wavelengths at which PL decays were measured. Reproduced with permission.^[^ ^70^ ^]^ Copyright 2016, IOP Publishing Ltd. c) Crown and PEABr co‐treated CsPbBr_3_ perovskite materials. i) PL for the perovskite films without (solid lines) or with (dashed lines) crown for different PEABr ratios. ii) PLQY (excited at 365 nm at ≈2.3 mW cm^−2^) versus PEABr ratio for the perovskite with and without crown. iii) TEM images for drop‐cast 40% PEABr perovskite precursor solution without (left) and with (right). Insets are the TEM images with small bars. Reproduced under the terms of the CC-BY 4.0 license.^[^ ^71^ ^]^ Copyright 2018, The Authors, published by Springer Nature.

Except for the key role in controlling the dimension of perovskite materials, the organic amines can also be used to adjust the perovskite size, following by the effective tune of emission wavelength.^[^ ^65^, ^66^ ^]^ By selecting appropriate organic and alkali metal salts, Worku et al.^[^ ^67^ ^]^ prepared light‐emitting thin films containing hollow perovskite NCs via facile solution processing. These perovskite NCs exhibit tunable PL emission wavelengths from green to blue under UV irradiation through varying the molar ratio of EDABr_2_ to the perovskite precursors (**Figure** **8**a‐i and Table 1). Simultaneously, a series of cyan‐emitting perovskites were also obtained with a maximum PLQY of around ≈55% (Figure 8a‐ii). As shown in Figure 8a‐iii, further blue shift emission from small quantum‐confined CsPbBr_3_ NCs could not be the case, because the average particle size of EDA5‐Na is much larger than the exciton Bohr radius of CsPbBr_3_ (≈7 nm). Therefore, these results suggested that the tunable PL emission with high PLQYs was due to the use of suitable organic and alkali metal salts. The organic cations (EDA^2+^) exhibited effective passivation ability for surface defects, whereas increasing the EDA^2+^ content will decrease grain sizes. Meanwhile, the small alkali metal cations (Na^+^) were helpful for the formation of hollow structures with the presence of large organic cations. As a result, quantum confinement could be realized in hollow perovskite NCs with controlled sizes and thicknesses.

For perovskite nanomaterials, the reaction temperature also shows strong effects on the as‐formed particle sizes. The particle size is highly related to the emission wavelengths and nonradiative recombination process. For example, according to the work from Song et al.,^[^ ^68^ ^]^ we found that the PL spectra of CsPbBr_3_ perovskite QDs could be tunable by merely changing the reaction temperature, resulting in the effective control of QDs sizes for hot injection. Besides, varying the size of the perovskite QDs and changing the halide component simultaneously are also effective in shifting the PL emission peak.^[^ ^69^ ^]^ As shown in Figure 8b‐i,ii, Ravi et al.^[^ ^70^ ^]^ prepared a series of CsPbBr_3_ nanocubes as well as nanoplatelets through adjusting the reaction temperatures in 2016. Consequently, the size of CsPbBr_3_ perovskites was control accurately, resulting in a tunable PL emission wavelength (blue→cyan→green). Moreover, the radiative lifetimes were found to be related to particle size too (Figure 8b‐iii). Specifically, the quantum confinement was increased though decreasing the average size of nanocubes, then, both the optical gap and the transition probability for radiative excitonic recombination were systematically enhanced, hence, the average radiative PL lifetime exhibited an obvious decrease with the increase in quantum confinement.

Another interesting work about the size engineering in cyan‐emitting perovskite materials was reported by Ban et al. in 2019.^[^ ^71^ ^]^ In that work, they used 1,4,7,10,13,16‐hexaoxacyclooctadecane (crown) and PEABr to decorate CsPbBr_3_ perovskite for controlling the domain size and film morphology. As shown in Figure 8c‐i, the PL emission spectrum displays blue shifts with increasing concentration of PEABr, which is ascribed to the decrease in the average perovskite crystals size. The authors declared that the PL peaks were gradually shifted from 520 nm (pale cyan, 0% PEABr) to 507 nm (cyan region, 100% PEABr). The PEABr incorporation also significantly increases the PL efficiencies (Figure 8c‐ii). Meanwhile, the addition of crown above 20% PEABr leads to a further enhancement of the PLQY (e.g., the 40% PEABr‐crown films exhibit a PLQY of 70%  ±  8%). Such a considerable enhancement can be attributed to the effective suppression of the nonradiative recombination processes. Furthermore, the crown's introduction into PEA‐based CsPbBr_3_ perovskite shows a controllable crystal size, resulting in tunable emission wavelength. According to this work, the following EL peaks of PeLEDs were also controllable at cyan emission (from 514 to 510 nm). Moreover, we notice that all‐inorganic copper‐based perovskite materials with cyan emission were prepared by Yang et al. in 2018.^[^ ^33^ ^]^ In that work, they used a ligand‐assisted reprecipitation technique to prepare these perovskites. Meanwhile, through the effective adjustment of particle sizes, the PL emissions at 385, 410, and 504 nm of Cs_2_CuCl_4_, Cs_2_CuBr_4_, and Cs_2_Cu(Br/I)_4_ QDs were achieved, respectively. In addition, the perovskite QDs emission region was further shifted to a deep blue range when the anion exchange was conducted. Unfortunately, such all‐inorganic copper‐based perovskite materials with cyan emission have not been further utilized to fabricate PeLEDs for investigating their EL performances. Similarly, the combination of quantum‐confined effect and quasi‐2D strategy was used for cyan‐emitting perovskite materials, and a high EQE (9.5%, emission at 483 nm, PLQY≥60%) was achieved.^[^ ^72^ ^]^

In this section, we have discussed various approaches for size engineering in cyan‐emitting perovskites, including the introduction of additive(s) and accurate control of reaction temperatures. It can be concluded that the quantum‐confined effect, the underlying mechanism of size engineering, is of great importance in tuning the emission wavelength and PLQY.

## 3 Perspectives

As summarized earlier, various strategies have been developed to obtain the cyan‐emitting metal halide perovskites, including compositional engineering, dimensional engineering, and size engineering. Compositional engineering is one of the most fundamental and important approaches for achieving colorful metal halide perovskite materials and devices. The rich preparation recipes are the first advantage for compositional engineering, because the A, B, and X sites do provide a magical combination. However, the modulation on the X site, especially for the mixed halogens, is often involved in the issues of phase instability. Therefore, the disordered halogen migration might be a serious obstruction for the further practical application of these materials. Dimensional engineering is a recently emerged promising strategy to blue shift the emission wavelengths of most metal halide perovskites. However, its practical application is still hampered by poor lattice stability and conductivity. The interaction of spacer (e.g., PEABr) and inorganic octahedron is not strong enough, leading to poor stability in low‐dimensional domains. Moreover, the long‐chained additives reduce the conductivity of perovskite emitter layer, resulting in a limiting performance for PeLEDs. As for size engineering, it usually requires exact control on particle size. However, as reported, the particle size changes over time, and particle size changes sometimes come with morphology evolution (nanocube, nanorod, and nanoplatelets). As a result, the PL emission properties become unstable. Therefore, to prepare high‐performance and stable perovskite materials and devices, a scientific combination of the above‐mentioned three approaches is highly encouraged.

Though cyan‐emitting perovskites have developed rapidly and achieved many notable results in recent years, there still exist many challenges. First, the mechanistic understanding of the above‐mentioned three classes of preparation strategies is somewhat limited. Moreover, a series of scientific issues regarding stability, structure–performance relationships, and intrinsic mechanisms for optical/electronic properties remain unresolved. Therefore, there is much‐needed urgency for the development of a theory‐guided deep understanding of cyan‐emitting perovskite. Meanwhile, the cyan‐emitting perovskite materials with various excellent properties simultaneously are also strongly encouraged. Another great challenge that we have to face is how to improve the overall performance of electrical PeLEDs devices while keeping a stable cyan emission.

As for the future development of preparing cyan‐emitting perovskite materials, we think that Pb‐free perovskite materials are encouraged. Pb‐based perovskites suffer from easy decomposition under humid or redox environments, resulting in significant degradation of performance and a potential hazard for humans and the environment. Hence, investigating efficient methods to stabilize the desired perovskite phase and increase the redox, humidity, and chemical resistance of Pb‐based perovskites is encouraged. Besides, we think that finding suitable techniques for effective recovery/reuse of Pb from perovskites is also meaningful. Moreover, we believe that the REEs will play a prominent role in shaping cyan‐emitting perovskites, including the Pb‐free perovskites, and the further improvements of optical/electric properties. Meanwhile, the optimized combination of multiple strategies is of great potential for making significant progress in developing high‐performance and stable cyan‐emitting perovskite materials.

In the end, we firmly believe that the cyan‐emitting perovskites will be widely used in the future, particularly in lighting and display. The white LEDs with a higher color‐rendering index could be achieved by introducing cyan‐emitting perovskite materials into the conventional device, such as GaN LEDs and yellow phosphor. Besides, the underwater light communication system may also be a potential application for cyan‐emitting perovskites with effective encapsulation technology. Specifically, by taking advantage of the low absorption of seawater, the cyan PeLEDs may play as a light source in underwater optical communication. These will facilitate the advance of cyan‐emitting perovskite materials and their applications in important optical and electric areas.

## 4 Conclusion

In this minireview, the preparation strategies of cyan‐emitting perovskites are summarized as compositional engineering, dimensional engineering, and size engineering. Compositional engineering, especially for metal ions doping, is beneficial to improve the performances of both perovskite materials and devices. The unique properties (e.g., enhanced stability and tunable emission) can also be achieved through dimensional engineering. The quantum‐confined effect in cyan‐emitting perovskite could be tunable by introducing suitable additive or accurate control of reaction temperatures. Overall, we believe that the high‐efficiency and stable cyan‐emitting perovskite materials and LEDs will be achieved shortly, and they will exhibit great potential in practical applications.

## Conflict of Interest

The authors declare no conflict of interest.
